# New records of bees of the genus *Sphecodes* Latreille in the Palaearctic part of China (Hymenoptera, Halictidae)

**DOI:** 10.3897/zookeys.792.28042

**Published:** 2018-10-23

**Authors:** Yulia V. Astafurova, Maxim Yu. Proshchalykin, Ze-qing Niu, Chao-dong Zhu

**Affiliations:** 1 Zoological Institute, Russian Academy of Sciences, Universitetskaya Nab., 1, Saint Petersburg 199034, Russia Zoological Institute, Russian Academy of Sciences Saint Petersburg Russia; 2 Federal Scientific Centre for East Asian Terrestrial Biodiversity, Far Eastern Branch of Russian Academy of Sciences, Vladivostok 690022, Russia Federal Scientific Centre for East Asian Terrestrial Biodiversity, Far Eastern Branch of Russian Academy of Sciences Vladivostok Russia; 3 Key Laboratory of Zoological Systematics and Evolution, Institute of Zoology, Chinese Academy of Sciences, 1 Beichen West Road, Chaoyang District, Beijing, 100101, PR China Institute of Zoology, Chinese Academy of Sciences Beijing China; 4 College of Life Sciences, University of Chinese Academy of Sciences, No.19(A) Yuquan Road, Shijingshan District, Beijing, 100049, PR China University of Chinese Academy of Sciences Beijing China

**Keywords:** Anthophila, Apiformes, cleptoparasites, fauna, new synonymy, taxonomy

## Abstract

The available information about the cleptoparastic bees of the genus *Sphecodes* in the Palaearctic part of China is summarized. Twenty-four species are currently known from this area including 16 newly recorded. Based on type specimens, new synonymies have been proposed for *Sphecodescristatus* Hagens, 1882 = *S.alfkeni* Meyer, 1922, **syn. n.**; *S.longulus* Hagens, 1882 = *S.subfasciatus* Blüthgen, 1934, **syn. n.**; *S.nippon* Meyer, 1922 = *S.kansuensis* Blüthgen, 1934, **syn. n.**; *Sphecodespieli* Cockerell, 1931 = *S.orientalis* Astafurova & Proshchalykin, 2014, **syn. n.** Lectotypes are designated for *Sphecodesalfkeni* Meyer, 1922 and *S.pellucidusniveipennis* Meyer, 1925. Illustrated keys to males and females of all species known from Palaearctic China and an updated checklist of the 33 Chinese species of *Sphecodes* are provided.

## Introduction

The present paper is part of a series of works dealing with the bees of the cleptoparastic genus *Sphecodes* Latreille, 1804 from the Palaearctic region (Astafurova and Proshchalykin 2014, 2015a, b, 2016a, b, 2017a, b, Astafurova et al. 2014, 2015, 2018). Consequently, we focus on species in northern China and do not deal with the southern, Oriental species of China.

The question of where the zoogeographical boundary exists between the Oriental and the Palaearctic regions in China has been discussed by many researchers working on various groups of animals (Emeljanov 1974, Hoffman 2001, Fellowes 2006, Chen et al. 2008, Heiser and Schmitt 2013, He et al. 2017). In this paper, the views of Pesenko (2007) and Astafurova (2013) are followed for halictid bees, which posit that the approximate border between Palaearctic and Oriental Regions in China lies between 30°–35° northern latitude.

*Sphecodesformosanus* Cockerell was the first species of the genus described from China (Taiwan) (Cockerell 1911). Since then, a total of ten species and three subspecies have been described (Meyer, four species and two subspecies; Cockerell, four species; Blüthgen, two species and one subspecies), seven of which are still valid (see section on taxonomy for details). Sixteen *Sphecodes* species have been recorded from China so far (Meyer 1920, 1922, 1925, Blüthgen 1924, 1927, 1934, Cockerell 1911, 1922, 1931, Strand and Yasumatsu 1938, Ascher and Pickering 2018). Among them, only seven species were known from the Palaearctic.

Based on a comprehensive study of specimens in various collections, we catalogue 24 species of the genus *Sphecodes*, with 16 species recorded from China for the first time. New synonymies are proposed for four specific names: *Sphecodescristatus* Hagens, 1882 = *S.alfkeni* Meyer, 1922, syn. n.; *S.longulus* Hagens, 1882 = *S.subfasciatus* Blüthgen, 1934, syn. n.; *S.nippon* Meyer, 1922 = *S.kansuensis* Blüthgen, 1934, syn. n.; *Sphecodespieli* Cockerell, 1931 = *S.orientalis* Astafurova & Proshchalykin, 2014, syn. n. Illustrated keys to the species known from the Palaearctic part of China are presented to facilitate further studies.

## Materials and methods

The results presented in this paper are based on 453 specimens collected in the Palaearctic part of China and currently housed in the Institute of Zoology, Chinese Academy of Sciences (Beijing, China, IZCAS); the Zoological Institute, Russian Academy of Sciences (St. Petersburg, Russia, ZISP); and the private collection of Maximilian Schwarz (Ansfelden, Austria, PCMS). The following acronyms are used for the collections where type specimens are deposited:

**MNHB**Museum für Naturkunde der Humboldt Universität zu Berlin, Germany.

**NHRS**Naturhistoriska riksmuseet, Stockholm, Sweden.

**USNM**National Museum of Natural History, Smithsonian Institution, Washington, DC, USA.

**KUF**Kyushu University, Fukuoka, Japan.

The taxonomy and distribution of species generally follow that of Warncke (1992), Bogusch and Straka (2012), and Astafurova and Proshchalykin (2017b). A detailed current synonymy of the species has been given by Astafurova and Proshchalykin (2017b). Morphological terminology follows that of Michener (2007) and Engel (2001). The ventral surface of some flagellomeres bear a distinctive patch of sensilla trichodea A (sensu Årgent and Svensson 1982), which we refer to as “tyloids;” they are easily observable under light microscopy. The abbreviations F, T, and S are used for flagellomere, metasomal tergum, and metasomal sternum, respectively. The density of integumental punctures is described using the following formula: puncture diameter (in μm) / ratio of distance between punctures to average puncture diameter, e.g., 15–20 μm / 0.5–1.5. Integumental sculpturing, aside from distinctive surface punctation, is described as follows: reticulate: superficially net-like or made up of a network of raised lines; rugose: irregular, non-parallel, wrinkled raised lines (rugae); tessellate: regular network of shallow grooves with flat interspaces.

Specimens were studied with a Leica M205A stereomicroscope and photographs were taken with a combination of a stereomicroscope (Olympus SZX10) and a digital camera (Canon EOS70D). Final images represent a composite of several photographs taken at different focal planes and combined using the program Helicon Focus 6. All images were post-processed for contrast and brightness using Adobe® Photoshop®.

The species are presented alphabetically and those that could not be inspected in this paper are quoted from published sources. We use the following abbreviations for collectors: **JH** – Jiri Halada, **PK** – Petr Kozlov; **VR** – Vsevolod Roborovsky. New distributional records are noted with an asterisk (*).

Unfortunately, we have not examined the type of *S.manchurianus*, because it was not found in Kyushu University (Japan). We also have not found specimens corresponding to the original description of Strand and Yasumatsu (1938) in our material.

## Taxonomy

### Key to the *Sphecodes* species of the Palaearctic part of China

Additional species are included in this key because they are widespread in the Palaearctic and may also occur in China. These include *S.maruyamanus* Tsuneki, 1983, *S.murotai* Tsuneki, 1983, *S.tanoi* Tsuneki, 1983 (known from Japan and Russian Far East), *S.miniatus* Hagens, 1882, *S.puncticeps* Thomson, 1870, and *S.reticulatus* Thomson, 1870. *Sphecodesmanchurianus* Strand & Yasumatsu, 1938, known only from the holotype, is not included.

### Males

**Table:** 

1	Costal margin of hind wing with 7–14 hamuli. Base of gonocoxite dorsally without impression. Usually large species: total body length 5.0–12.0 mm	**2**
–	Costal margin of hind wing with 5–6 hamuli. Base of gonocoxite dorsally with or without impression. Large or small species: total body length 3.5–11.0 mm	**11**
2	Head rounded, about as long as wide. Hind wing with basal (*M*) vein strongly curved. T1 finely and sparsely (sometimes indistinctly) punctate. Gonostylus dorsally with small rectangular process directed to penis valve (Fig. 15). Body length 7.0–10.0 mm	***S.monilicornis* (Kirby)**
–	Head transverse, wider than long. Hind wing with basal (*M*) vein weakly curved. T1 distinctly coarsely and densely punctate. Gonostylus another shape	**3**
3	Mesoscutum densely punctate, with confluent punctures (areolate)	**4**
–	Mesoscutum sparsely punctate, medially with punctures separated by at least a puncture diameter	**5**
4	Head more transverse, 1.2 times as wide as long. Vertex long, distance from top of head to upper margin of lateral ocellus about 2.5–3.0 lateral ocellar diameters as seen in dorsal view. Tyloids on flagellomeres (at least from F4 onward) semicircular across basal 1/5–1/3 and linear across remaining flagellomeres as seen in lateral view. Mesoscutellum sparsely punctate, medially with punctures separated by more than a puncture diameter and often with impunctate areas. T1 completely red. Gonostylus larger, not narrowed apically (Fig. 1). Body length 9.0–12.0 mm	***S.albilabris* (Fabricius)**
–	Head less transverse, 1.1 times as wide as long. Vertex shorter, distance from top of head to upper margin of lateral ocellus about two lateral ocellar diameters as seen in dorsal view. Tyloids on flagellomeres semicircular across basal 1/6–1/4, linear portion along remaining flagellomeres not developed. Mesoscutellum densely punctate, with confluent punctures. T1 black or brownish at least on basal 1/3 Gonostylus smaller, distinctly narrowed apically (Fig. 23). Body length 7.0–12.0 mm	***S.scabricollis* Wesmael**
5	Vertex with a longitudinal carina. Gonostylus smaller, not overlapped apically, as in Figs 4, 21	**6**
–	Vertex without a longitudinal carina. Gonostylus larger, another shape, overlapped apically	**8**
6	Tyloids on flagellomeres (at least from F4 onward) are semicircular across basal 1/3–1/2. T1 with marginal zone very finely and indistinctly punctate. Body length 7.0–10.0 mm	***S.cristatus* Hagens**
–	Tyloids on flagellomeres weakly developed, very narrow, semicircular across basal 1/7–1/5 of flagellomere. T1 with marginal zone coarsely and distinctly punctate	**7**
7	Head more transverse, 1.2 times as wide as long. Mesoscutum more coarsely punctate (30–75 μm). T2 with marginal zone impunctate. Larger: total body length 7.0–11.0 mm	***S.olivieri* Lepeletier de Saint-Fargeau**
–	Head less transverse, 1.10–1.15 times as wide as long. Mesoscutum more finely punctate (25–40 μm). T2 with marginal zone distinctly punctate. Smaller: total body length 5.0–7.0 mm	***S.pectoralis* Morawitz**
8	Vertex long, distance from top of head to upper margin of lateral ocellus about three lateral ocellar diameters as seen in dorsal view. Tyloids on flagellomeres cover at least 1/3 part of flagellomere Gonostylus with long apical process (Fig. 9)	**9**
–	Vertex shorter, distance from top of head to upper margin of lateral ocellus about two lateral ocellar diameters as seen in dorsal view. Tyloids on flagellomeres not covering more than 1/4 part of flagellomere. Gonostylus another shape at tip, as in Fig. 2	**10**
9	Tyloids on flagellomeres well developed, covering large part of flagellomere (as seen in lateral view, Fig. 57). Body length 7.0–14.0 mm	***S.gibbus* (Linnaeus)**
–	Tyloids on flagellomeres weakly developed, covering about 1/3 of flagellomere (as seen in lateral view, Fig. 58). Body length 7.0–11.0 mm	***S.nippon* Meyer**
10	T4 with marginal zone finely tessellate, without punctures (Fig. 46). Body length 7.0–10.0 mm	***S.reticulatus* Thomson**
–	T4 with marginal zone distinctly punctate, smooth between punctures (rarely indistinctly tessellate) (Fig. 45). Body length 7.0–12.0 mm	***S.alternatus* Smith**
11	Base of gonocoxite dorsally without impression	**12**
–	Base of gonocoxite dorsally with impression	**19**
12	T1 densely punctate. Gonostylus elongate (Fig. 18). Body length 5.0–5.5 mm	***S.nurekensis* Warncke**
–	T1 impunctate or with a few fine punctures. Gonostylus another shape	**13**
13	Vertex coarsely and densely punctate, ocello-ocular area with confluent punctures, separated by at most a half puncture diameter	**14**
–	Vertex finely and sparser punctate, ocello-ocular area with punctures, separated by at least a puncture diameter	**17**
14	Vertex with longitudinal carina (in *S.kozlovi* usually weakly developed) (Figs 38, 39)	**15**
–	Vertex without longitudinal carina	**16**
15	Vertex with well visible longitudinal carina. Felt-like areas on last flagellomeres cover at least 1/2 underside of flagellomere, F2 as long as wide (Fig. 53). T1 impunctate or with a few fine punctures. Membranous portion of gonostylus smaller, as in Fig. 19. Body length 6.0–9.0 mm	***S.pieli* Cockerell**
–	Vertex with weakly visible longitudinal carina. Felt-like areas on last flagellomeres cover about 1/3 underside of flagellomere, F2 slightly longer than wide (Fig. 54). T1 sparsely, but coarsely punctate. Membranous portion of gonostylus large, as in Fig. 12. Body length 8.0–10.0 mm	***S.kozlovi* Astafurova & Proshchalykin**
16	Tyloids on last flagellomeres (at least from F4 onward) usually cover more than 1/2 of ventral flagellar surfaces, often up to 4/5 Membranous portion of gonostylus larger, as in *S.kozlovi* (Fig. 12). Body length 7.0–11.0 mm	***S.pellucidus* Smith**
–	Tyloids on last flagellomeres (at least from F4 onward) usually cover about 1/2 of ventral flagellar surfaces, rarely up to 3/4. Membranous portion of gonostylus smaller (Fig. 6). Body length 6.0–9.0 mm	***S.ephippius* (Linné)**
17	Ocello-ocular area densely punctate, with punctures separated by about one puncture diameter. Gonostylus joining apex and partly inner surface of gonocoxite (Fig. 22). Body length 5.0–7.5 mm	***S.puncticeps* Thomson**
–	Ocello-ocular area sparsely punctate, with punctures separated by 1–3 puncture diameters. Gonostylus joining only apex of gonocoxite (Fig. 8)	**18**
18	F2 shorter, 1.4–1.6 times as long as wide. Tyloids on the flagellomeres extend across 1/4–1/2 of ventral flagellar surfaces. Body length 3.5–6.0 mm	***S.longulus* Hagens**
–	F2 longer, 1.7–1.8 times as long as wide. Tyloids on the flagellomeres extend across 1/2–3/4 of ventral flagellar surfaces. Body length 4.0–5.0 mm	***S.turanicus* Astafurova & Proshchalykin**
19	T1 densely punctate. Face with appressed white pubescence below and above the antennal toruli	**20**
–	T1 impunctate or with sparse punctures (in *S.miniatus* sometimes relatively densely punctate). Face with appressed white pubescence only below the antennal toruli	**22**
20	Tyloids variable, covering 1/2–4/5 flagellar ventral surfaces. Membranous portion of gonostylus small, triangular (Fig. 17)	***S.schwarzi* Astafurova & Proshchalykin**
–	Tyloids covering from 3/4 to entire ventral flagellar surfaces. Membranous portion of gonostylus large, close to rectangular (Figs 10, 20)	**21**
21	Antenna longer, F2 1.4 times as long as wide (Fig. 55). Membranous portion of gonostylus almost straight on inner edge (Fig. 20). Body length 5.0–7.5 mm	***S.pinguiculus* Pérez**
–	Antenna shorter, F2 1.2 times as long as wide (Fig. 56). Membranous portion of gonostylus weakly *S*-curved on inner edge (Fig. 10). Body length 5.0–7.5 mm	***S.intermedius* Blüthgen**
22	Pronotum, between dorsal and lateral surfaces, rounded, not angulate (Fig. 36)	**23**
–	Pronotum, between dorsal and lateral surfaces, with sharp angle (Fig. 35)	**26**
23	Tyloids on flagellomeres covering less than 1/3 of ventral flagellar surfaces. Membranous portion of gonostylus larger, trapezoidal (Fig. 5). Body length 6.0–9.0 mm	***S.ferruginatus* Hagens**
–	Tyloids on flagellomeres (at least from F4 onward) covering about 1/2–3/4 or entire of ventral flagellar surfaces. Membranous portion of gonostylus smaller, oval or almost square (Figs 13, 16, 24)	**24**
24	Clypeus with fine, simple and sparsely plumose setae, sculpturing clearly visible (Fig. 33). Membranous portion of gonostylus square (Fig. 13). Body length 6.0–7.0 mm	***S.maruyamanus* Tsuneki**
–	Clypeus with densely plumose setae, partly obscuring sculpturing (Fig. 34). Membranous portion of gonostylus close to oval	**25**
25	Antenna short, middle flagellomeres as long as or slightly longer than wide. Tyloids on flagellomeres covering entire of ventral flagellar surfaces. Membranous portion of gonostylus longer, reach penis valve (Fig. 16). Body length 5.5–6.5 mm	***S.murotai* Tsuneki**
–	Antenna long, flagellomeres (from F3 onward) 1.2–1.3 times as long as wide. Tyloids on flagellomeres (at least from F4 onward) covering about 1/2–3/4 of ventral flagellar surfaces. Membranous portion of gonostylus shorter, not reach penis valve (Fig. 24). Body length 6.0–7.0 mm	***S.tanoi* Tsuneki**
26	F2 short, 0.9–1.0 times as long as F3. Tyloids on flagellomeres (at least from F4 onward) usually cover entire ventral flagellar surfaces. Gonostylus with trapezoidal membranous portion (Fig. 7). Body length 5.0–6.5 mm	***S.geoffrellus* (Kirby)**
–	F2 longer, 1.1–1.2 as long as F3. Tyloids on flagellomeres shorter, covering at most 4/5 the ventral flagellar surfaces (in *S.miniatus* tyloids on last flagellomeres rare cover entire ventral flagellar surfaces)	**27**
27	Tyloids on flagellomeres covering more than 3/4 flagellar ventral surfaces. Gonostylus with large, trapezoidal membranous portion (Fig. 14). Body length 4.0–6.0 mm	***S.miniatus* Hagens**
–	Felt-like areas on flagellomeres cover less 1/3 underside of flagellomere. Gonostylus with oval membranous portion or without one	**28**
28	Head less transverse, 1.05 times as wide as long. Mesoscutum sparsely punctate, medially with punctures mostly separated by 1–3 puncture diameters. T1–T3 usually red, rarely terga entirely black. Gonostylus with oval membranous portion (Fig. 3). Body length 5.0–7.0 mm	***S.crassus* Thomson**
–	Head more transverse, at least 1.15 times as wide as long. Mesoscutum very densely punctate, with confluent punctures (areolate). Terga usually wholly black, rare T1 dark red. Gonostylus without membranous portion (Fig. 11). Body length 7.5–8.5 mm	***S.laticaudatus* Tsuneki**

### Females

**Table:** 

1	Hind wing with basal (*M*) vein weakly curved; costal margin with 7–14 hamuli. Usually large species: total body length 6.0–15.0 mm	**2**
–	Hind wing with basal (*M*) vein strongly curved; costal margin with 5–6 hamuli. Large or small species: total body length 5.5–11.0 mm	**10**
2	Vertex less elevated (distance from top of head to upper margin of lateral ocellus less two lateral ocellar diameters as seen in frontal view), with longitudinal sharp carina (Fig. 37)	**3**
–	Vertex more elevated (distance from top of head to upper margin of lateral ocellus more two lateral ocellar diameters as seen in frontal view), acarinate, but sometimes with weak (indistinct) longitudinal ridge	**5**
3	Face and gena with sparse, semi-erect, gray pubescence not obscuring integument. T1 with finer punctures (3–10 μm). Body length 6.0–8.0 mm	***S.cristatus* Hagens**
–	Face and gena with dense, appressed, snow-white pubescence obscuring integument. T1 with coarser punctures (10–30 μm)	**4**
4	Mesoscutum coarsely punctate (25–75 μm). T2 with marginal zone impunctate. Larger: body length 8.0–11.0 mm	***S* . *olivieri* Lepeletier de Saint Fargeau**
–	Mesoscutum relatively finely punctate (25–40 μm). T2 with marginal zone distinctly punctate. Smaller: body length 6.5–8.5 mm	***S.pectoralis* Morawitz**
5	Gena flat. Preoccipital lateral carina developed (Fig. 40). Body length 9.0–12.0 mm	***S.scabricollis* Wesmael**
–	Gena swollen. Preoccipital carina not developed	**6**
6	Vertex shorter, distance from top of head to upper margin of lateral ocellus about 2 lateral ocellar diameters as seen in dorsal view (Fig. 42). T4 with marginal zone punctate and smooth between punctures or finely tessellate without punctures	**7**
–	Vertex longer, distance from top of head to upper margin of lateral ocellus equal to 2.5–3.0 lateral ocellar diameters as seen in dorsal view (Fig. 41). T4 with marginal zone impunctate, smooth (rarely indistinctly tessellate)	**8**
7	T4 with marginal zone impunctate, finely tessellate (Fig. 46); T1 finely punctate (10–15 μm). Mesoscutum usually densely punctate, medially with punctures separated by not more than 1–3 puncture diameters, sometimes sparser. Body length 7.0–10.0 mm	***S.reticulatus* Thomson**
–	T4 with marginal zone distinctly punctate, smooth between punctures (rarely indistinctly tessellate) (Fig. 45); T1 coarsely punctate (15–25 μm). Mesoscutum usually sparsely punctate, medially with punctures separated by mostly 2–4 puncture diameters. Body length 8.0–11.0 mm	***S.alternatus* Smith**
8	Mesoscutum densely punctate, with punctures separated by less than a puncture diameter (Fig. 47). Body length 9.0–12.0 mm	***S.albilabris* (Fabricius)**
–	Mesoscutum sparsely punctate, medially with punctures separated by at least 2 puncture diameters (Fig. 50)	**9**
9	Head rounded-rectangular on upper margin, square-shaped as seen in frontal view (Fig. 32); vertex sparsely punctate, punctures mostly separated by more than a puncture diameter. Pygidial plate equal or slightly narrower than metabasitarsus. T1 usually indistinctly punctate, with a few very fine punctures. Body length 7.0–10.0 mm	***S.monilicornis* (Kirby)**
–	Head uniformly rounded on upper margin, oval as seen in frontal view (Fig. 28); vertex densely punctate, punctures mostly separated by less than a puncture diameter. Pygidial plate 0.5–0.6 times as wide as metabasitarsus. T1 distinctly punctate, with fine and coarser punctures. Body length 7.0–15.0 mm	***S.gibbus* (Linnaeus) and *S.nippon* Meyer**
	Females of this vicarious species are very difficult to distinguish morphologically; however, *S.nippon* is distributed in China to Gansu on the West, whereas *S.gibbus* is distributed in China to Xinjiang on the East.	
10	Mandible simple (without an inner tooth)	**11**
–	Mandible bidentate	**13**
11	Head narrower, at most 1.15 times as wide as long (Fig. 26). Body length 4.0–6.0 mm	***S.longulus* Hagens**
–	Head broader, 1.2–1.3 times as wide as long	**12**
12	Face, gena and mesepisternum with gray, sparse, semi-erect pubescence, not obscuring integument. Metasoma coarsely punctate (10–15 μm). Pygidial plate as wide as metabasitarsus. Body length 5.0–8.0 mm	***S.puncticeps* Thomson**
–	Face, gena and mesepisternum with dense, snow-white, appressed, pubescence obscuring integument (Fig. 31). Metasoma finely punctate (3–5 μm). Pygidial plate 1.2 times as wide as metabasitarsus	***S.turanicus* Astafurova & Proshchalykin**
13	Pygidial plate at least 1.2 times wider than metabasitarsus, usually dull. Mesoscutum densely punctate, punctures usually separated by less than two puncture diameters. Total body length 7.0–11.0 mm	**14**
–	Pygidial plate equal to or narrower than metabasitarsus, shiny. Mesoscutum usually sparsely punctate, disc medially with punctures separated by more than two puncture diameters. Total body length 4.0–9.0 mm	**18**
14	Vertex with longitudinal carina (Figs 38, 39)	**15**
–	Vertex without longitudinal carina	**16**
15	Vertex with obvious longitudinal carina (Fig. 38). Setae on scape shorter than width of scape. Pygidial plate 1.2–1.4 times wider than metabasitarsus. Body length 7.0–9.0 mm	***S.pieli* Cockerell**
–	Vertex with weakly visible longitudinal carina (Fig. 39). Setae on scape distinctly longer than width of scape (not obviously in old females). Pygidial plate 1.4–1.5 times wider than metabasitarsus. Body length 8.0–9.0 mm	***S.kozlovi* Astafurova & Proshchalykin**
16	Pygidial plate 1.6–1.7 times as wide as metabasitarsus. Gena wider, 0.8 times as wide as eye in lateral view. Mesoscutum densely punctate, with punctures separated by at most a puncture diameter (Fig. 51). Body length 7.5–10.0 mm	***S.laticaudatus* Tsuneki**
–	Pygidial plate 1.2–1.5 times as wide as metabasitarsus. Gena narrower, 0.7 times as wide as eye in lateral view. Mesoscutum sparsely punctate, medially with punctures usually separated by 1–2 puncture diameters (Fig. 52)	**17**
17	Head more transverse, 1.30–1.35 times as wide as long; vertex, behind ocelli, not elevated in frontal view. Setae on scape distinctly longer than width of scape. Pygidial plate 1.3–1.5 times as wide as metabasitarsus	***S.pellucidus* Smith**
–	Head less transverse, 1.20–1.25 times wider than long; vertex, behind ocelli, weakly elevated. Setae on scape shorter than width of scape. Pygidial plate 1.2–1.4 times as wide as metabasitarsus	***S.ephippius* (Linné) and *S.grahami* Cockerell**
	Females of these species are very difficult to distinguish morphologically; however, *S.ehippius* is distributed in North-West China (Xinjiang), whereas *S.grahami* is recorded from Central, South and East China; the male of *S.grahami* is unknown	
18	Clypeus densely punctate, punctures separated by less than one puncture diameter. Pronotum, between dorsal and lateral surfaces, rounded, not angulate (Fig. 36)	**19**
–	Clypeus sparsely punctate, punctures separated by at least one puncture diameter. Pronotum, between dorsal and lateral surfaces, with sharp angle (Fig. 35)	**22**
19	Hind femur narrow, regularly pointed toward distal end, its length more than 3.5 times its maximum width. Body length 6.0–7.5 mm	***S.maruyamanus* Tsuneki**
–	Hind femur widened in proximal half, its length at most 3 times its maximum width	**20**
20	Vertex, behind ocelli, weakly elevated in frontal view (Fig. 25). Body length 6.0–9.0 mm	***S.ferruginatus* Hagens**
–	Vertex not elevated in frontal view (Fig. 30)	**21**
21	Thorax ventrally with sculpture finer that on sides (Fig. 44). Pygidial plate slightly narrower than metabasitarsus. Body length 6–7 mm	***S.tanoi* Tsuneki**
–	Thorax ventrally with sculpture as coarse as that on sides (Fig. 43). Pygidial plate as wide as metabasitarsus. Body length 5.5–6.5 mm	***S.murotai* Tsuneki**
22	Vertex longer, distance from top of head to upper margin of lateral ocellus equal to about 3–3.5 times lateral ocellar diameters as seen in dorsal view. Upper half of gena with appressed, dense pubescence obscuring integument	**23**
–	Vertex shorter, distance from top of head to upper margin of lateral ocellus equal to about two lateral ocellar diameters as seen in dorsal view. Gena with erect, sparse pubescence	**24**
23	Mesoscutum and mesoscutellum very sparsely punctate, with tiny punctures separated by 1–7 diameters (Fig. 48). Body length 5.0–7.0 mm	***S.pinguiculus* Pérez**
–	Mesoscutum and mesoscutellum more densely punctate, with coarse punctures separated by 1–3 puncture diameters (Fig. 49). Body length 6.5–8.5 mm	***S.intermedius* Blüthgen**
24	F3 transverse, 0.6–0.7 times as long as wide, as long as F1. Pygidial plate 0.9–1.0 as wide as metabasitarsus	**25**
–	F3 square, as long as wide, longer than F1 Pygidial plate 0.6–0.8 as wide as metabasitarsus	**26**
25	Paraocular area with dense, strongly plumose setae below the antennal toruli (Fig. 29). Body length 4.5–5.5 mm	***S.schwarzi* Astafurova & Proshchalykin**
–	Face with sparse, simple and weakly plumose setae (Fig. 27). Body length 4.0–6.0 mm	***S.miniatus* Hagens**
26	Head more transverse, 1.25 times as wide as long. Labrum trapezoidal, 0.7 times as long as wide. Hind femur strongly enlarged on proximal half, maximum width 0.4 times its length. Body length 5.0–8.0 mm	***S.crassus* Thomson**
–	Head less transverse, 1.1 times as wide as long. Labrum semicircular, 0.5 times as long as width. Hind femur weakly enlarged on proximal half, maximum width 0.35 times its length. Body length 4.5–6.5 mm	***S.geoffrellus* (Kirby)**

(Females of this vicarious species are very difficult to distinguish morphologically; however, *S.nippon* is distributed in China to Gansu on the West, whereas *S.gibbus* is distributed in China to Xinjiang on the East).

(Females of these species are very difficult to distinguish morphologically; however, *S.ehippius* is distributed in North-West China (Xinjiang), whereas *S.grahami* is recorded from Central, South and East China; the male of *S.grahami* is unknown).

## List of species

### Sphecodes albilabris

Taxon classificationAnimaliaHymenopteraHalictidae

(Fabricius, 1793)

Figs 1 , 47

#### Material examined.

CHINA: *Liaoning*, 1 ♀, 50 km N Mukden [Shenyang] [42°12'N, 123°23'E], 20.VII.1952, leg. Rubtsov (ZISP); *Inner Mongolia*, 1 ♂, Xilinhot [43°54'N, 116°00'E], 27.VII.1971, leg. Y.-W. Zhang (IZCAS); *Hebei*, 1 ♀, Kreis Yongnian [36°25'N, 114°14'E], 1995, leg. S.-Q. Li (IZCAS); 1 ♀, Yangjiaping [39°58'N, 115°24'E], 3.VIII.1937, leg. O. Piel (IZCAS); 1 ♀, Yu Xian, Xiheying [39°57'N, 114°00'E], 800 m, 29.VII.1964, leg. B.-Q. Li (IZCAS); *Beijing*, 1 ♀, Xiyuan [39°55'N, 116°24'E], 50 m, 23.VII.1962, leg. C.-G. Wang (IZCAS); *Shanxi*, 1 ♀, Xiexian, Zhongtiao Shan Mts. [34°48'N, 111°36'E], 22–24.V.1996, leg. JH (PCMS); *Gansu*, 1 ♂, Lanzhou [36°00'N, 103°25'E], 1500 m, 9.IX.1957, leg. Y.-R. Zhang (IZCAS).

#### Distribution.

*China (Liaoning, Inner Mongolia, Hebei, Beijing, Shanxi, Gansu), Central Asia, Russia, Europe (north to Finland and Sweden), Turkey, Syria, Caucasus, North Africa, Israel, India.

### Sphecodes alternatus

Taxon classificationAnimaliaHymenopteraHalictidae

Smith, 1853

Figs 2 , 46

#### Material examined.

CHINA: *Gansu*, 2 ♂♂, oasis Sachjou [Dunhuang] [40°09'N, 94°40'E], Gashun Gobi desert, 1–3.VIII.1895, VR, PK (ZISP); *Xinjiang*, 1 ♂, Yining, Boro Hqro Mts [44°06'N, 81°00'E], 27.VII.1991, Snizek (PCMS); 1 ♀, 37 ♂♂, Bugas near Khami [43°14'N, 93°50'E], 20.VIII–8.IX.1895, VR, PK (ZISP).

#### Distribution.

*China (Gansu, Xinjiang), Central Asia, Europe, Russia (south of European part and east to Khakassia Republic), Turkey, Iran, North Africa.

### Sphecodes crassus

Taxon classificationAnimaliaHymenopteraHalictidae

Thomson, 1870

Figure 3

#### Material examined.

CHINA: *Inner Mongolia*, 2 ♀♀, Suburgan-gol, Alashan [Helan Shan] Mt., Gobi, 29–30.VI.1908, PK (ZISP); 6 ♀♀, Tszosto, Alashan [Helan Shan] Mt., Gobi, 13–14.V.1908, PK (ZISP); *Shanxi*, 1 ♀, Xiexian, Zhongtiao Shan Mts [34°48'N, 111°36'E], 22–24.V.1996, leg. JH (PCMS); 2 ♀♀, 13 km S Yichuan [35°54'N, 110°36'E], 19.V.1996, leg. JH (PCMS).

#### Distribution.

*China (Inner Mongolia, Shanxi), Central Asia, Mongolia, Russia, Europe (north to 64°), Caucasus, Turkey, Iran, Japan, North Africa.

### Sphecodes cristatus

Taxon classificationAnimaliaHymenopteraHalictidae

Hagens, 1882

Figure 4

Sphecodes alfkeni Meyer, 1922: 172, ♀ (lectotype: ♀, **designated here**, China, Tientsin [Tianjin], [leg.] Weber, MNHB, examined). – Syn. n.

#### Material examined.

CHINA: *Heilongjiang*, 1 ♀, Harbin [45°46'N, 126°39'E], 8.X.1952 (IZCAS); 1 ♀, idem, 27.VII.1952 (IZCAS); 1 ♀, idem, 19.VII.1953 (IZCAS); 1 ♀, idem, 25.VII.1955 (IZCAS); 1 ♂, idem, 8.X.1952 (IZCAS); 1 ♂, idem, 16.VII.1952 (IZCAS); *Jilin*, 1 ♀, Gongzhuling [43°79'N, 124°69'E], 9.VI.1962, leg. T.-L. Cheng (IZCAS); *Liaoning*, 1 ♂, 50 km N Mukden [Shenyang], 20.VII.1952, Rubtsov (ZISP); 1 ♀, Guicheng [43°40'N, 126°15'E], 30.VII.1962, leg. T.-L. Cheng (IZCAS); *Inner Mongolia*, 4 ♂♂, 2 ♀♀, Dingyuanying [Bayan Hot], Alashan [Helan Shan] Mt., 22.V., 26.V., 17–26.IX.1908, PK (ZISP); 1 ♂, Tszosto, Alashan [Helan Shan] Mt., Gobi, 13–14.V.1908, PK (ZISP); 1 ♂, Ulanqab Men, Tomortei [41°48'N, 113°06'E], 31.VIII.1971 (IZCAS); 2 ♂♂, Hailar Shi [49°12'N, 119°42'E], 3.VIII.2006, leg. H.-Y. Zhang (IZCAS), 2 ♂♂, idem, 2.VIII.2006, leg. Y.-J. Li (IZCAS), 1 ♂, idem, 8.VIII.2006, leg. P. Wang (IZCAS); 2 ♂♂, Ordos, Dundatu [37°43'N, 108°10'E], 24.VII.2006, leg. Y.-J. Li (IZCAS); 2 ♀♀, 1 ♂, idem, 25.VII.2006, leg. P. Wang (IZCAS), 5 ♀♀, 3 ♂♂, idem, 25.VII.2006, leg. H.-Y. Zhang (IZCAS); 3 ♀♀, idem, 25.VII.2006, leg. M. Luo (IZCAS); 1 ♀, 1 ♂, Hohhot Shi, Heling Xian, Mengniu Zheng [40°49'N, 111°39'E], 15.VII.2006, leg. Y.-J. Li (IZCAS); 1 ♂, Hulun Buir Meng, Manzhouli Shi [49°12'N, 119°45'E], 5.VIII.2006, leg. Y.-J. Li (IZCAS); 1 ♀, Uxin Qi, Batugou [38°38'N, 108°53'E], 28.VII.2006, leg. M. Luo (IZCAS); *Hebei*, 1 ♀, Yangyuan Xian, Liumafang [40°11'N, 114°28'E], 950 m, 12.IV.2002, leg. Z.-Q. Niu (IZCAS); *Tianjin*, 1 ♀, Balitai [38°57'N, 117°19'E], 13.X.1953, leg. Z.-R. Yu (IZCAS). *Beijing*, 1 ♀, 1 ♂, Xiangshan [39°54'N, 116°12'E], 100 m, 19.IX.1962, leg. Y.-S. Shi (IZCAS); 3 ♂♂, Beijing [39°55'N, 116°24'E], 28.VIII.1973, leg. S.-F. Wang (IZCAS); 1 ♀, Wofosi [40°03'N, 115°10'E], 100 m, 10.V.1962, leg. S.-Y. Wang (IZCAS); 1 ♂, idem, 18.IX.1981, leg. Q. Zhou (IZCAS); 1 ♀, Zizhuyuan [39°57'N, 116°19'E], 24.IV.1962, leg. S.-M. Ge (IZCAS); *Shanxi*, 1 ♀, Qingjian env. [36°54'N, 110°36'E], 15.V.1996, leg. JH (PCMS); 1 ♀, Monan [34°42'N, 111°42'E], 26–28.V.1996, leg. JH (PCMS); 1 ♀, Suide, [37°18'N, 110°42'E], 13–14.V.1996, leg. JH (PCMS); *Shandong*, 1 ♀, Jinan [36°48'N, 117°01'E], 24.VI.1937 (IZCAS); *Shaanxi*, 1 ♀, Gangui [36°48'N, 110°18'E], 35 km NE Yanan, 17–18.V.1996, leg. JH (PCMS); *Ningxia*, 1 ♂, Ningxia [Yinchuan], Ordos, Gobi, 1–4.VI.1908, PK (ZISP); 6 ♂♂, Yanchi Xian, Sidunzi [37°28'N, 107°09'E], 1455 m, 22.VI.2016, leg. Z.-Q. Niu, D. Zhang (IZCAS); *Xinjiang*, 1 ♀, Jimsar Xian [44°00N 89°03E], 14.V.1955, leg. S.-J. Ma, K.-L. Xia, Y.-L. Cheng (IZCAS); 1 ♀, Jeminay Xian, S229, 14 km [47°14'N, 85°19'E], 1080 m, 28.VIII.2002, leg. Z.-Q. Niu (IZCAS); 1 ♀, Tian Shan [43°10'N, 86°00'E], 28.VIII.1957, leg. G. Wang (IZCAS); 1 ♀, Manas Xian, Shihezi [44°07'N, 86°00'E], 550 m, 6.VI.1957, leg. C.-P. Hong (IZCAS); 1 ♀, idem, 590 m, 27.VIII.1957, leg. S.-Y. Wang (IZCAS); 1 ♀, Yining Xian [44°00'N, 81°21'E], 4.VIII.1957, leg. W.-Y. Yang (IZCAS).

#### Published records.

Blüthgen 1927: 42, as *Sphecodesalfkeni* Meyer (Tianjin); Ascher and Pickering 2018 (Beijing).

#### Distribution.

China (*Heilongjiang, *Jilin, *Liaoning, *Inner Mongolia, *Hebei, Tianjin, Beijing, *Shanxi, *Shandong, *Shaanxi, *Ningxia, *Xinjiang), Europe (north to Sweden), Korea, Russia, Caucasus, Turkey, Central Asia, Mongolia.

### Sphecodes ephippius

Taxon classificationAnimaliaHymenopteraHalictidae

(Linné, 1767)

Figure 6

#### Material examined.

CHINA: *Xinjiang*, 1 ♂, Yaerkate [42°52'N, 92°50'E], 3.VIII.1956, leg. W.-Y. Yang (IZCAS).

#### Distribution.

*China (Xinjiang), Russia (east to Irkutsk Prov.), Mongolia, Central Asia, Caucasus, Turkey, Europe (north to 62°).

### Sphecodes ferruginatus

Taxon classificationAnimaliaHymenopteraHalictidae

Hagens, 1882

Figs 5 , 25 , 36

#### Material examined.

CHINA: *Beijing*, 2 ♀♀, 3 ♀♀, Bada Ling, Sanbu [40°22'N, 115°58'E], 500 m, 18, 27.IV.2002, leg. Z.-Q. Niu (IZCAS); 1 ♀, Miaofengshan [40°01'N, 115°59'E], 24.V.1957, leg. M.-H. Wang (IZCAS); 1 ♀, Wofosi [40°03'N, 115°10'E], 15.V.1961, leg. S.-M. Ge (IZCAS); *Shanxi*, 1 ♀, Xiexian [34°48'N, 111°36'E], Zhongtiao Shan Mts., 22–24.V.1996, leg. JH (PCMS).

#### Distribution.

*China (Beijing, Shanxi), Central Asia, Russia, Europe (north to 66°), Caucasus, Turkey, Japan.

### Sphecodes geoffrellus

Taxon classificationAnimaliaHymenopteraHalictidae

(Kirby 1802)

Figure 7

#### Material examined.

CHINA: *Heilongjiang*, 1 ♂, Da Hinggan Ling [51°42'N, 126°36'E], 23.VII.1980 (IZCAS); *Shaanxi*, 11 ♀♀, Gangui [36°48'N, 110°18'E], 35 km NE Yanan, 17–18.V.1996, leg. JH (PCMS); 1 ♀, 13 km S Yichuan [35°54'N, 110°36'E], 19.V.1996, leg. JH (PCMS).

#### Distribution.

*China (Heilongjiang, Shanxi), Central Asia, Europe (north to 66°), Russia (east to Far East), Turkey, Near East, Mongolia, Japan.

### Sphecodes gibbus

Taxon classificationAnimaliaHymenopteraHalictidae

(Linnaeus, 1758)

Figs 9 , 41 , 57

Sphecodes gibbus var. turkestanicus Meyer, 1920: 113 (holotype: 1 ♀, Uzbekistan: Golodnaja Steppe [Gulistan], MNHB). Synonymized by Blüthgen 1923: 510.

#### Material examined.

CHINA: *Xinjiang*, 1 ♀, 13 ♂♂, Bugas near Khami [43°14'N, 93°50'E], 20.VIII–8.IX.1895, leg. VR, PK (ZISP); 1 ♂, Qitai Xian [44°31'N, 90°06'E], 10.VII.1975 (IZCAS); 1 ♀, Kashi [39°14'N, 75°32'E], 133 m, 10.VII.1959, leg. C.-Q. Li (IZCAS); 1 ♂, idem, 10.VII.1959, leg. A-F. Tian (IZCAS).

#### Published records.

Meyer, 1920: Yarkand (Xinjiang), as Sphecodesgibbusvar.turkestanicus Meyer, 1920.

#### Distribution.

China (Xinjiang), Central Asia, Russia (east to Yakutia), Pakistan, Mongolia, Europe (north to 63°), Turkey, Israel, North Africa, India.

### Sphecodes grahami

Taxon classificationAnimaliaHymenopteraHalictidae

Cockerell, 1922

Figure 52

Sphecodes grahami Cockerell, 1922: 12 (holotype: ♀, China, Sichuan: Suifu, Graham coll.; USNM);

#### Material examined.

CHINA: *Shanxi*, 1 ♀, Xiexian [34°48'N, 111°36'E], Zhongtiao Shan Mts., 22–24.V.1996, leg. JH (PCMS); *Shaanxi*, 2 ♀♀, Gangui [36°48'N, 110°18'E], 35 km NE Yanan, 17–18.V.1996, leg. JH (PCMS).

#### Published records.

Cockerell 1922: 12 (Shanghai); Ascher and Pickering 2018 (Jilin, Hebei, Anhui, Jiangsu, Shanghai, Zhejiang, Yunnan, Xizang, Guandong).

#### Distribution.

China (Jilin, Hebei, *Shanxi, *Shaanxi, Anhui, Jiangsu, Shanghai, Shanghai, Zhejiang, Sichuan, Yunnan, Xizang, Guandong).

#### Remark..

The female of this species is challenging to distinguish from West-Palaearctic *S.ephippius* (Linné, 1767).

### Sphecodes intermedius

Taxon classificationAnimaliaHymenopteraHalictidae

Blüthgen, 1923

Figs 10 , 49 , 56

#### Material examined.

CHINA: *Gansu*, 1 ♂, Shibendu, oasis Sachjou [Dunhuang] [40°09'N, 94°40'E], Gashun Gobi desert, 9–12.VIII.1895, leg. VR, PK (ZISP).

#### Distribution.

*China (Gansu), Central Asia, Europe, Russia (European part, Ural), North Africa, Caucasus, Turkey.

### Sphecodes kozlovi

Taxon classificationAnimaliaHymenopteraHalictidae

Astafurova & Proshchalykin, 2015

Figs 12 , 39 , 54

#### Material examined.

CHINA: *Inner Mongolia*, 3 ♀♀, Tszosto, Alashan [Helan Shan] Mt., Gobi, 10–18.V.1908, leg. PK (ZISP); 7 ♀♀, Dingyuanying [Bayan Hot], Alashan [Helan Shan] Mt., 10, 18–19.VI.1908, 15–16. IV.1909, leg. PK (ZISP); *Shanxi*, 1 ♀, Monan [34°42'N, 111°42'E], 26–28.V.1996, leg. JH (PCMS); *Ningxia*, 2 ♀♀, Yanchi [37°24'N, 107°36'E], 11.V.1996, leg. JH (PCMS).

#### Distribution.

*China (Inner Mongolia, Shanxi, Ningxia), Mongolia (Dornod Aimag, Khentii Aimag).

### Sphecodes laticaudatus

Taxon classificationAnimaliaHymenopteraHalictidae

Tsuneki, 1983

Figs 11 , 51

#### Material examined.

CHINA: *Hebei*, 1 ♂, Xinglong Xian, Wuling Shan [40°26'N, 117°31'E], 28.VIII.1973 (IZCAS).

#### Distribution.

*China (Hebei), Russia (Far East), Japan.

### Sphecodes longulus

Taxon classificationAnimaliaHymenopteraHalictidae

Hagens, 1882

Figs 8 , 26

Sphecodes subfasciatus Blüthgen, 1934: 22, ♀ (holotype: ♀, China, S. Kansu, 19.VI.1930, leg. Hummel, NHRS, examined). – Syn. n.

#### Material examined.

CHINA: *Inner Mongolia*, 1 ♀, Goytzo valley, Alashan, Gobi, 5.IV.1908, leg. PK (ZISP); *Hebei*, 1 ♀, Changli Xian [39°38'N, 119°05'E], 28.IV.1962, leg. T.-L. Cheng (IZCAS); 1 ♀, Xiaowutai Shan [38°36'N, 115°39'E], 1200 m, 22.VIII.1964, leg. Y.-H. Han (IZCAS); *Shaanxi*, 1 ♀, Gangui [36°48'N, 110°18E‘], 35 km NE Yanan, 17–18.V.1996, leg. JH (PCMS); *Gansu*, 1 ♀, Lanzhou [36°00'N, 103°25'E], 27.IV.1955, leg. S.-J. Ma, K.-L. Xia, Y.-L. Cheng (IZCAS); *Xinjiang*, 1 ♂, Tacheng Xian [46°25'N, 82°32'E], 470 m, 10.IX.1960, leg. S.-Y. Wang (IZCAS); 1 ♂, Bostanterak [39°07'N, 95°03'E], 9.VII.1959, leg. S.-Y. Wang (IZCAS).

#### Distribution.

China (*Inner Mongolia, *Hebei, *Shaanxi, Gansu, *Xinjiang), Central Asia, Russia, Europe (north to Finland, Sweden, Denmark, England), Turkey, Syria, Japan.

### Sphecodes manchurianus

Taxon classificationAnimaliaHymenopteraHalictidae

Strand & Yasumatsu, 1938

Sphecodes manchurianus Strand & Yasumatsu, 1938: 80 (holotype: ♂, China : “Fengtien (Mukden), South Manchoukuo, 5.VIII.1930, T. Nozawa leg.”; KUF, lost).

#### Material examined.

No material examined.

#### Distribution.

China (Liaoning).

#### Remark.

Known only from the holotype.

### Sphecodes monilicornis

Taxon classificationAnimaliaHymenopteraHalictidae

(Kirby, 1802)

Figs 15 , 32

#### Material examined.

CHINA: *Heilongjiang*, 1 ♂, Morin Dawa [47°21'N, 128°03'E], 24.VII.1976 (IZCAS); 1 ♂, Harbin [45°46'N, 126°39'E], 6.VII.1947 (IZCAS); 1 ♀, idem, 27.VII.1952 (IZCAS); 2 ♂♂, idem, 25.VII.1953 (IZCAS); 7 ♂♂, idem, 4.VII.1955 (IZCAS); 1 ♂, idem, 8.VII.1955 (IZCAS); 1 ♀, 3 ♂♂, idem, 10.VII.1955 (IZCAS); 1 ♀, 5 ♂♂, idem, 19.VII.1955 (IZCAS); 5 ♂♂, idem, 10.VII.1955 (IZCAS); 1 ♂, idem, 11.VII.1955 (IZCAS); 3 ♂♂, idem, 23.VII.1953 (IZCAS); 1 ♂, idem, 9.VIII.1955 (IZCAS).

#### Distribution.

*China (Heilongjiang), Central Asia, Mongolia, Russia, North Pakistan, Europe (north to 64°), Caucasus, Turkey, North Africa.

### Sphecodes nippon

Taxon classificationAnimaliaHymenopteraHalictidae

Meyer, 1922

Figs 28 , 50 , 58

Sphecodes kansuensis Blüthgen, 1934: 21, fig. 11, ♂ (holotype: ♂, China, S. Kansu [Gansu] 19.VI.1930, leg. Hummel, NHRS, examined). – Syn. n.

#### Material examined.

CHINA: *Heilongjiang*, 1 ♂, Harbin [45°46'N, 126°39'E], 24.IX.1950; 1 ♂, idem, 16.VII.1952; 1 ♂, idem, 25.VII.1952; 1 ♂, idem, 23.VII.1953; 1 ♂, idem, 11.VII.1954; 1 ♂, idem, 4.VII.1955; 4 ♂♂, idem, 8.VII.1955; 2 ♂♂, idem, 25.VII.1955 (IZCAS); 2 ♂♂, Mao’ershan [47°21'N, 128°03'E], 29.VII.1951 (IZCAS); 1 ♂, Hengdaohezi [45°57'N, 129°57'E], 28.VII.1951 (IZCAS); *Inner Mongolia*, 1 ♂, 3 ♀♀, Dingyuanying [Bayan Hot], Alashan [Helan Shan] Mt., 16–17.V., 3–6.VI., 11–16.IX.1908, PK (ZISP); *Hebei*, 1 ♂, Yangjiaping [39°58'N, 115°24'E], 17.VII.1937; 1 ♂, idem, 20.VII.1937; 1 ♀, idem, 6.VII.1937; 1 ♀, idem, 8.VII.1937, leg. O. Piel (IZCAS); 1 ♂, Xiaowutai Shan [38°36'N, 115°39'E], 1200 m, 25.VIII.1964; 1 ♂, idem, 11.VII.1964; 1 ♂, idem, 12.VII.1964, leg. Y.-H. Han (IZCAS); 1 ♂, Xinglong Xian, Wuling Shan [40°26'N, 117°31'E], 28.VIII.1973 (IZCAS); *Tianjin*, 1 ♀, Balitai [38°57'N, 117°19'E], 24.IV.1953, leg. Z.-Y. Xu (IZCAS); *Beijing*, 1 ♂, 40 km N Beijing [40°09'N, 116°14'E], 28.IX.1952, Rubtsov (ZISP); 1 ♀, Bada Ling [40°22'N, 115°58'E], 700 m, 2.VII.1974, leg. Y.-S. Shi (IZCAS); 1 ♀, Bada Ling, Sanbu [40°22'N, 115°58'E], 500 m, 27.IV.2002, leg. Z.-Q. Niu (IZCAS); 1 ♀, Xidazhuangke village, Songshan [40°31'N,115°47'E], 910 m, 15.V.2007, leg. H.-R. Huang (IZCAS); 1 ♀, Miaofengshan [40°01'N, 115°59'E], 2.VIII.1957; 1 ♂, idem, 18.VII.1957, leg. M.-H. Wang (IZCAS); *Shaanxi*, 9 ♀♀, Gangui [36°48'N, 110°18'E], 35 km NE Yanan, 17–18.V.1996, leg. JH (PCMS); *Gansu*, 5 ♂♂, oasis Sachjou [Dunhuang] [40°09'N, 94°40'E], Gashun Gobi desert, 28–30.VII.1895, leg. VR, PK; 1 ♀, Lanzhou, 25.VII.1908, leg. PK (ZISP).

#### Distribution.

China (*Heilongjiang, *Inner Mongolia, *Hebei, *Tianjin, *Beijing, *Shaanxi, Gansu), Russia (East Siberia, Far East), Mongolia, Japan.

### Sphecodes nurekensis

Taxon classificationAnimaliaHymenopteraHalictidae

Warncke, 1992

Figure 18

#### Material examined.

CHINA: *Xinjiang*, 1 ♂, Bugas near Khami [N43°14’ E93°50’], 20.VIII.1895, leg. VR, PK (ZISP); 1 ♂, Ürümqi [43°28'N, 87°32'E], 980 m, 2.IX.1959, leg. S.-Y. Wang (IZCAS).

#### Distribution.

*China (Xinjiang), Tajikistan.

### Sphecodes olivieri

Taxon classificationAnimaliaHymenopteraHalictidae

Lepeletier de Saint-Fargeau, 1825

Figs 21 , 37

#### Material examined.

CHINA: *Gansu*, 1 ♀, 2 ♂♂, oasis Sachjou [Dunhuang] [40°09'N, 94°40'E], Gashun Gobi desert, 24.VII, 1–3.VIII.1895, leg. VR, PK (ZISP); 1 ♂, Zhangye [38°32'N, 100°14'E], 1450 m, 29.VII.1957, leg. Y.-R. Zhang (IZCAS); *Xinjiang*, 117 ♂♂, Bugas near Khami [43°14'N, 93°50'E], 20.VIII-8.IX.1895, leg. VR, PK (ZISP); 3 ♀♀, Manas Xian [44°10'N, 86°07'E], 400 m, 9.VI.1953, leg. C.-P. Hong (IZCAS); 1 ♀, idem, 9.VI.1953, leg. W.-Y. Yang (IZCAS); 1 ♂, Manas Xian, Shihezi [44°07'N, 86°00'E], 500 m, 27.VIII.1959, leg. C.-Q. Li (IZCAS); 1 ♂, Burqin Xian [47°25'N, 86°32'E], 480 m, 25.VIII.1960, leg. S.-Y. Wang (IZCAS); 1 ♂, Turpan Xian [42°32'N, 89°07'E], 30.VI.1958 (IZCAS).

#### Distribution.

*China (Gansu, Xinjiang), Central Asia, South Europe, Russia (south of European part), Caucasus, Turkey, Iran, Pakistan, India, Israel, United Arab Emirates, Qatar, North Africa.

### Sphecodes pectoralis

Taxon classificationAnimaliaHymenopteraHalictidae

Morawitz, 1876

#### Material examined.

CHINA: *Gansu*, 1 ♀, 1 ♂, oasis Sachjou [Dunhuang] [40°09'N, 94°40'E], Gashun Gobi desert, 28.VII–4.VIII.1895, leg. VR, PK (ZISP); *Xinjiang*: 4 ♀♀, 140 ♂, Bugas near Khami [43°14'N, 93°50'E], 20.VIII–8.IX.1895, leg. VR, PK (ZISP).

#### Distribution.

*China (Gansu, Xinjiang), Central Asia.

### Sphecodes pellucidus

Taxon classificationAnimaliaHymenopteraHalictidae

Smith, 1845

Figure 35

Sphecodes pellucidus var. hybridus Blüthgen, 1924: 516, ♀ (syntypes: ♀♀, China: Sichuan, NHRS). Synonymized by Warncke 1992: 20.
Sphecodes pellucidus var. niveipennis Meyer, 1925: 7, ♂ (lectotype: ♂, **designated here**, Chin. Turkestan, Uss-Lusch. Jarkand [China, Xinjiang, Yarkand] 1600 m, 4–6.8.90, Conrandt S. / pellucidus v. niveipennis Dr. R Meyer det.; MNHB, examined). Synonymized by Warncke 1992: 20.

#### Material examined.

CHINA: *Gansu*, 1 ♀, Lanzhou, 11–25.III.1901, leg. PK (ZISP); 3 ♂♂, Dankhe River, S to Sachzhou [Dunhuang], Gashunskoe Gobi [39°55'N, 94°20'E], 24.VII.1895, leg. VR, PK (ZISP).

#### Distribution.

China (*Gansu, Xinjiang, Sichuan), Central Asia, Russia, Mongolia, Europe (north to 66°), Caucasus, Turkey, North Africa.

### Sphecodes pieli

Taxon classificationAnimaliaHymenopteraHalictidae

Cockerell, 1931

Figs 19 , 38 , 53

Sphecodes pieli Cockerell, 1931: 13, ♂ (holotype: ♂, China, Shanghai, Zo-Se, June 16, 1930 (Piel No 34), USNM).
Sphecodes orientalis Astafurova & Proshchalykin, 2014: 517–518, ♀, ♂ (holotype: ♂, Russia, Primorskiy Terr.: 15 km SW Slavyanka, 31.VIII.1995, S. Belokobylskij, ZISP, examined). – Syn. n.

#### Material examined.

CHINA: *Hebei*, 1 ♀, Xiaowutai Shan [38°36'N, 115°39'E], 1200 m, 19.VI.1964, leg. Y.-H. Han (IZCAS); *Beijing*, 1 ♀, Bada Ling, Sanbu [40°22'N, 115°58'E], 500 m, 27.IV.2002 (IZCAS); 2 ♀♀, idem, 28.IV.2002, leg. Z.-Q. Niu (IZCAS); 1 ♀, Xidazhuangke village, Songshan [40°31'N, 115°47'E], 910 m, 15.V.2007, leg. H.-R. Huang (IZCAS); 1 ♀, Mentougou, Xiaolongmen, Liyuanling [39°58'N, 115°28'E], 1140–1250 m, 19.V.2002, leg. Z.-Q. Niu (IZCAS); *Shaanxi*, 1 ♀, Gangui, 35 km NE Yanan [36°48'N, 110°18'E], 17–18.V.1996, leg. JH (PCMS); 1 ♀, 13 km S Yichuan [35°59'N, 110°36'E], 19.V.1996, leg. JH (PCMS); 1 ♀, Jingangling, 50 km W Linfen, [36°12'N, 111°42'E], 29–30.V.1996, leg. JH (PCMS); *Sichuan*, 1 ♀, Nanping, Ta Zang [33°15'N, 104°15'E], 2200 m, 15–18.VI.1990, JH (PCMS).

#### Published records.

Ascher and Pickering, 2018 (Zhejiang, Jiangsu).

#### Distribution.

China (*Hebei, *Beijing, *Shaanxi, Jiangsu, Shanghai, Zhejiang, *Sichuan), Russia (Far East).

### Sphecodes pinguiculus

Taxon classificationAnimaliaHymenopteraHalictidae

Pérez, 1903

Figs 20 , 48 , 55

#### Material examined.

CHINA: *Inner Mongolia*, 1 ♀, Dingyuanying [Bayan Hot], Alashan [Helan Shan] Mt., 22–24.VI.1908, PK (ZISP); *Gansu*, 1 ♀, oasis Sachjou [Dunhuang] [40°09'N, 94°40'E], Gashun Gobi desert, 4.VIII.1895, leg. VR, PK (ZISP).

#### Distribution.

*China (Inner Mongolia, Gansu), Central Asia, Mongolia, Russia, South Europe, Caucasus, Turkey, Iran, Israel, United Arab Emirates, North Africa, Cape Verde Islands.

### Sphecodes scabricollis

Taxon classificationAnimaliaHymenopteraHalictidae

Wesmael, 1835

Figs 23 , 40

#### Material examined.

CHINA: *Heilongjiang*, 2 ♀♀, Harbin [45°46'N, 126°39'E], 11.VII.1954 (IZCAS); 1 ♀, idem, 8.VII.1955 (IZCAS); 1 ♀, idem, 19.VII.1955 (IZCAS); 1 ♀, idem, 20.VII.1955 (IZCAS); 2 ♀♀, idem, 25.VII.1955 (IZCAS); 1 ♂, idem, 8.VII.1955 (IZCAS), 2 ♂♂, idem, 25.VII.1955 (IZCAS); 1 ♂, idem, 11.IX.1955 (IZCAS); *Liaoning*, 1 ♂, Guicheng [43°40'N, 126°15'E], 10.VII.1962, leg. T.-L. Cheng (IZCAS); *Beijing*, 1 ♂, Changping district, Liucun town, Wangjiayuan village [40°12'N, 116°00'E], 5.IX.2011, leg. F. Yuan (IZCAS); 16 ♂♂, Wofosi [40°03'N, 115°10'E], 18.IX.1981, leg. Y.-R. Wu (IZCAS); 2 ♂♂, idem, 10.IX.1981, leg. Q. Zhou (IZCAS); 2 ♀♀, idem, 10.IX.1981, leg. W.-Z. Ma (IZCAS); 1 ♀, Bada Ling [40°22'N, 115°58'E], 3.IX.1981, leg. P.-Y. Yu (IZCAS); 1 ♂, idem, 28.VIII.1974 (IZCAS); 1 ♂, 13.VIII.1981 (IZCAS); 1 ♂, 20.VIII.1988, leg. Y.-S. Shi (IZCAS); 1 ♂, idem, 30.VIII.1977, leg. S.-F. Wang (IZCAS); 1 ♂, idem, 25.VIII.1981, leg. Q. Zhou (IZCAS); 1 ♀, idem, 7.IX.1982, leg. Z.-C. Jin (IZCAS); 1 ♂, Xiangshan [39°54'N, 116°12'E], 22.IX.1983, leg. J.-G. Fan (IZCAS); 4 ♀♀, Qinglongqiao [39°54'N, 116°21'E], 5.IX.1988, leg. H.-L. Xu (IZCAS); 1 ♂, idem, 12.V.1981, leg. Y.-R. Wu (IZCAS); 4 ♀♀, 1 ♂, Beijing [39°55'N, 116°24'E], 28.VIII.1973, leg. Y.-R. Wu (IZCAS); 3 ♂♂, idem, 28.VIII.1973, leg. S.-F. Wang (IZCAS); *Shaanxi*, 2 ♀♀, Qihling Mts., 6 km E Xunyangba [33°32'N, 108°33'E], 1000–1300 m, 23.V–13.VI.1998, leg. Marshal (PCMS); *Qinghai*, 1 ♂, Sinin-khe River valley [36°30'N, 101°40'E], 29.VII.1908, leg. PK (ZISP); *Zhejiang*, 1 ♂, Lian Country, West Tianmu Mt. [30°20'N, 119°25'E], 1000 m, 16.IX.2000, S. Belokobylskij (ZISP).

#### Distribution.

*China (Heilongjiang, Liaoning, Beijing, Shaanxi, Qinghai, Zhejiang), Kazakhstan (East Kazakhstan), Russia, Europe (north to S England and Latvia), Caucasus, Turkey, Iran, South Korea, Japan, India.

### Sphecodes turanicus

Taxon classificationAnimaliaHymenopteraHalictidae

Astafurova & Proshchalykin, 2017

Figure 31

#### Material examined.

CHINA: *Gansu*, 1 ♀, 2 ♂♂, oasis Sachjou [Dunhuang] [40°09'N, 94°40'E], Gashun Gobi desert, 1–9.VIII.1895, leg. VR, PK (ZISP).

#### Distribution.

*China (Gansu), Central Asia.

**Figures 1–12.**
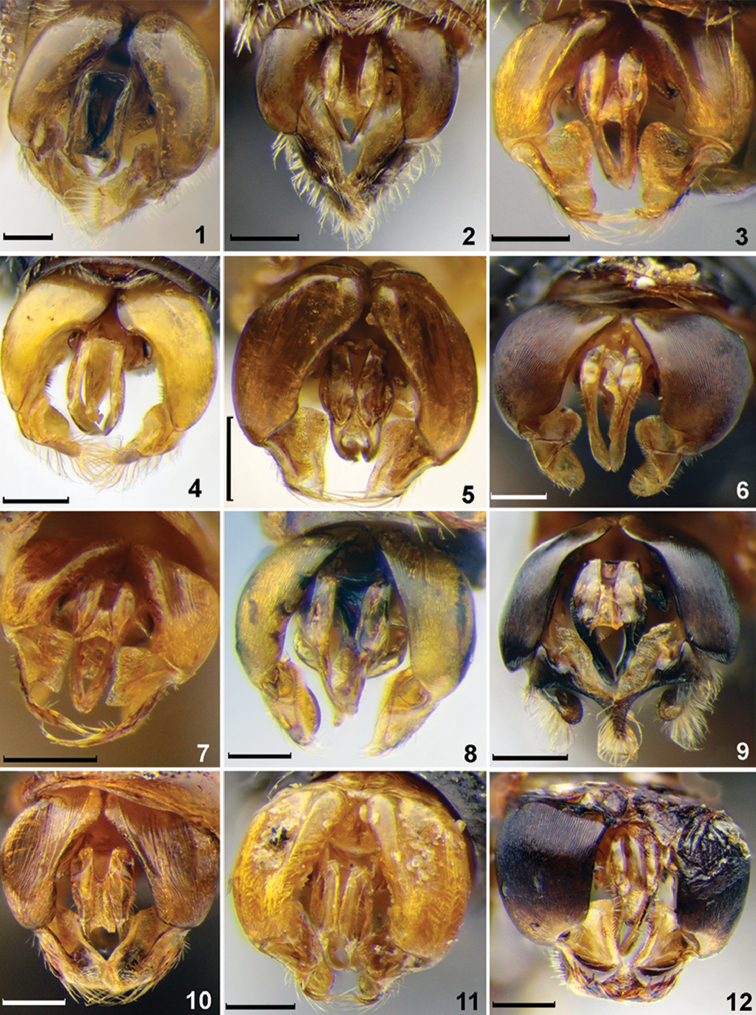
Genitalia, males, dorsal view. **1***Sphecodesalbilabris* (Fabricius) **2***S.alternatus* Smith **3***S.crassus* Thomson **4***S.cristatus* Hagens **5***S.ferruginatus* Hagens **6***S.ephippius* (Linné) **7***S.geoffrellus* (Kirby) **8***S.longulus* Hagens **9***S.gibbus* (Linnaeus) **10***S.intermedius* Blüthgen **11***S.laticaudatus* Tsuneki **12***S.kozlovi* Astafurova & Proshchalykin. Scale bars: 0.25 mm.

**Figures 13–24.**
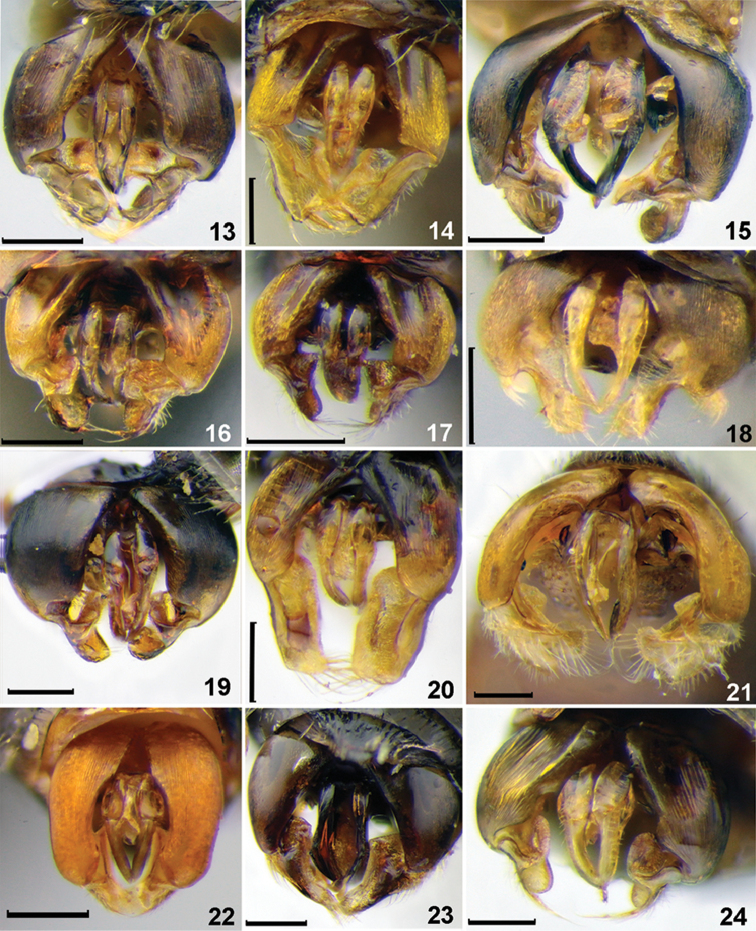
Genitalia, males, dorsal view. **13***Sphecodesmaruyamanus* Tsuneki **14***S.miniatus* Hagens **15***S.monilicornis* (Kirby) **16***S.murotai* Tsuneki **17***S.schwarzi* Astafurova & Proshchalykin **18***S.nurekensis* Warncke **19***S.pieli* Cockerell **20***S.pinguiculus* Pérez **21***S.olivieri* Lepeletier de Saint Fargeau **22***S.puncticeps* Thomson **23***S.scabricollis* Wesmael **24***S.tanoi* Tsuneki. Scale bars: 0.25 mm.

**Figures 25–30.**
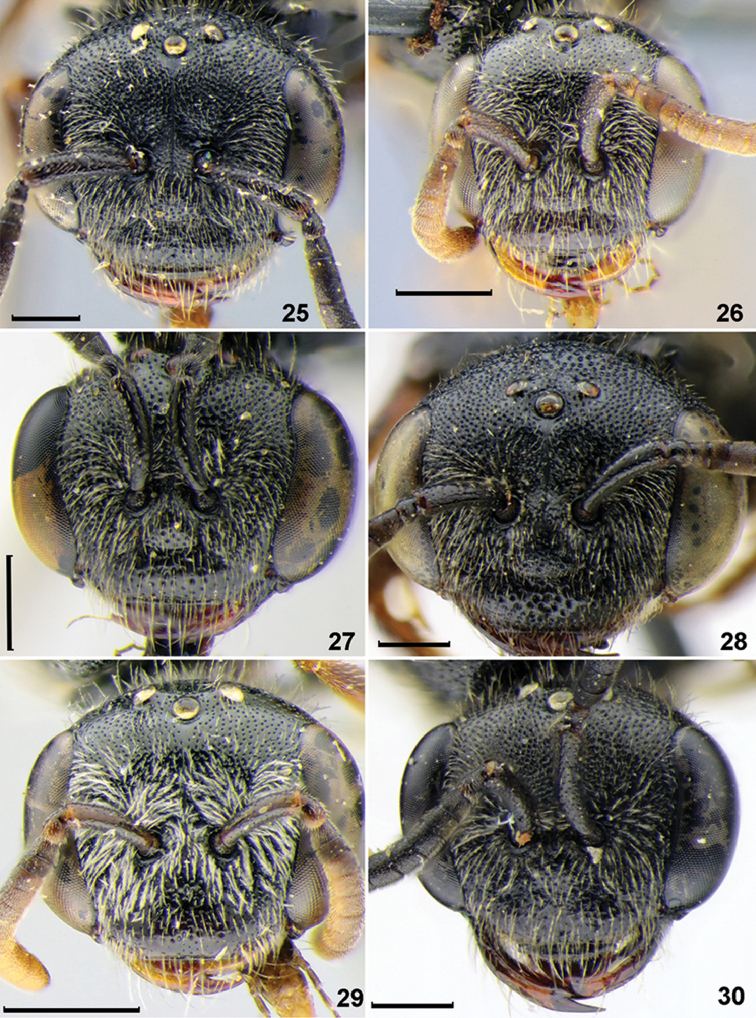
Head, females, frontal view. **25***Sphecodesferruginatus* Hagens **26***S.longulus* Hagens **27***S.miniatus* Hagens **28***S.nippon* Meyer **29***S.schwarzi* Astafurova & Proshchalykin **30***S.tanoi* Tsuneki. Scale bars: 0.5 mm.

**Figures 31–36.**
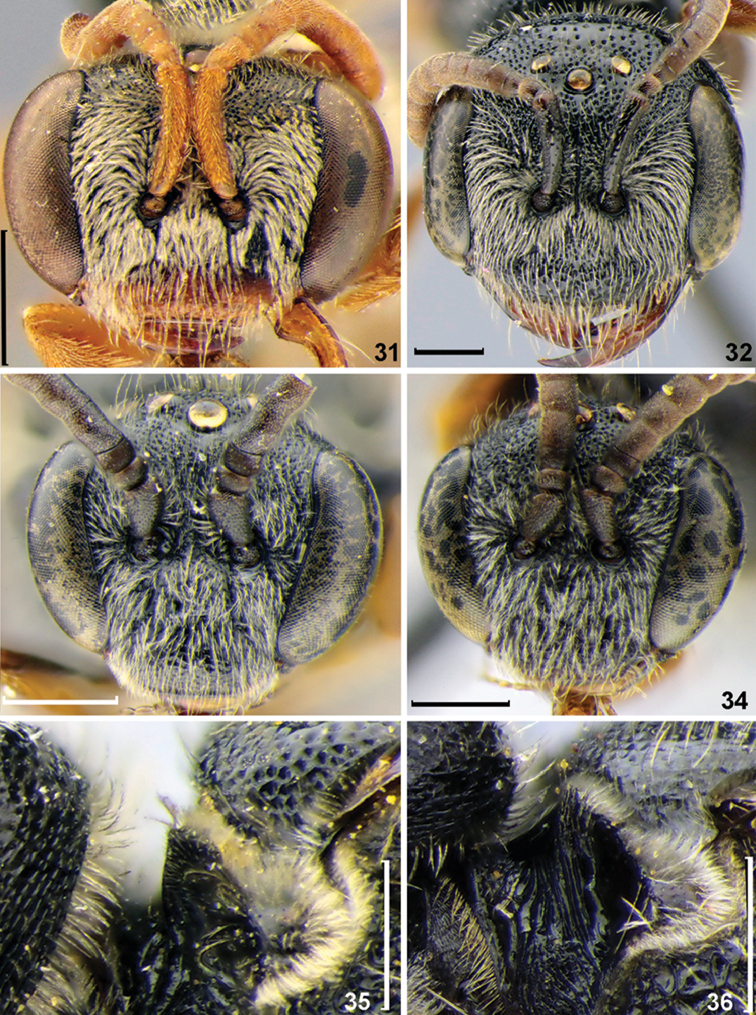
Diagnostic characters of *Sphecodes* species. **31, 32, 35, 36** Females **33, 34** Males **31–34** head, frontal view **35, 36** pronotum, lateral, view **31***Sphecodesturanicus* Astafurova & Proshchalykin **32***S.monilicornis* (Kirby) **33***S.maruyamanus* Tsuneki **34***S.murotai* Tsuneki **35***S.pellucidus* Smith **36***S.ferruginatus* Hagens. Scale bars: 0.5 mm.

**Figures 37–40.**
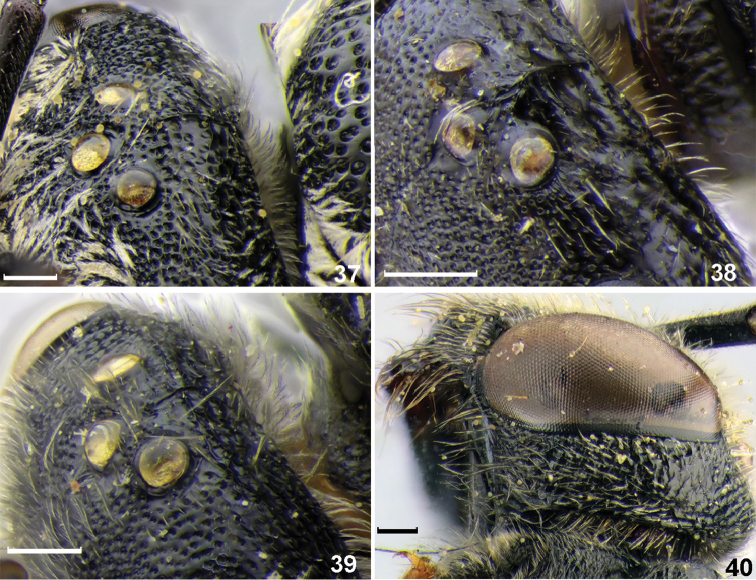
Diagnostic characters of *Sphecodes* species, females. **37–39** vertex, dorso-lateral view **40** preoccipital carina and gena, dorso-lateral view **37***Sphecodesolivieri* Lepeletier de Saint Fargeau **38***S.pieli* Cockerell **39***S.kozlovi* Astafurova & Proshchalykin **40***S.scabricollis* Wesmael. Scale bars: 0.25 mm.

**Figures 41–46.**
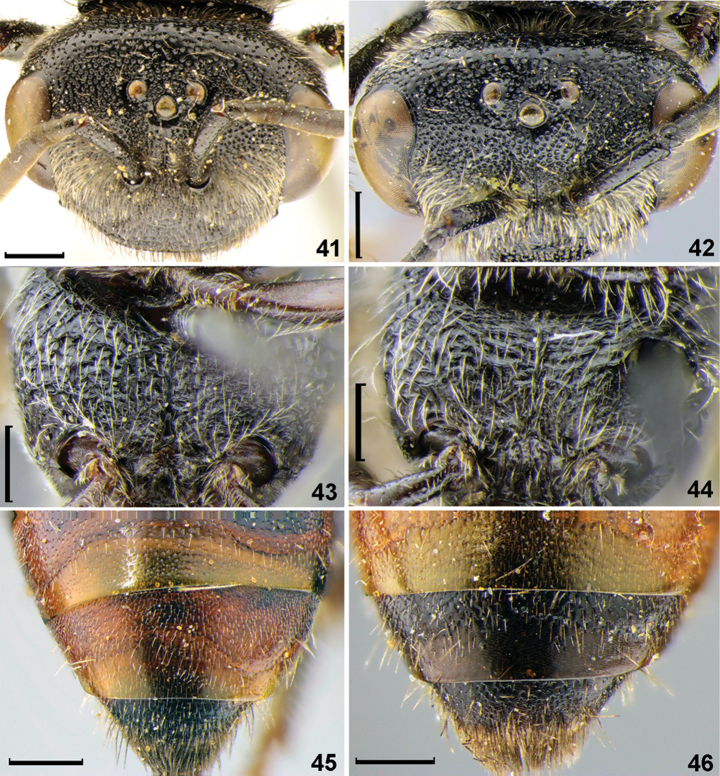
Diagnostic characters of *Sphecodes* species, females. **41, 42** head, dorsal view **43, 44** thorax, ventral view **45, 46** T4-T5, dorsal view **41***S.gibbus* (Linnaeus) **42, 45***S.reticulatus* Thomson **43***S.murotai* Tsuneki **44***S.tanoi* Tsuneki **46***S.alternatus* Smith. Scale bars: 0.5 mm.

**Figures 47–52.**
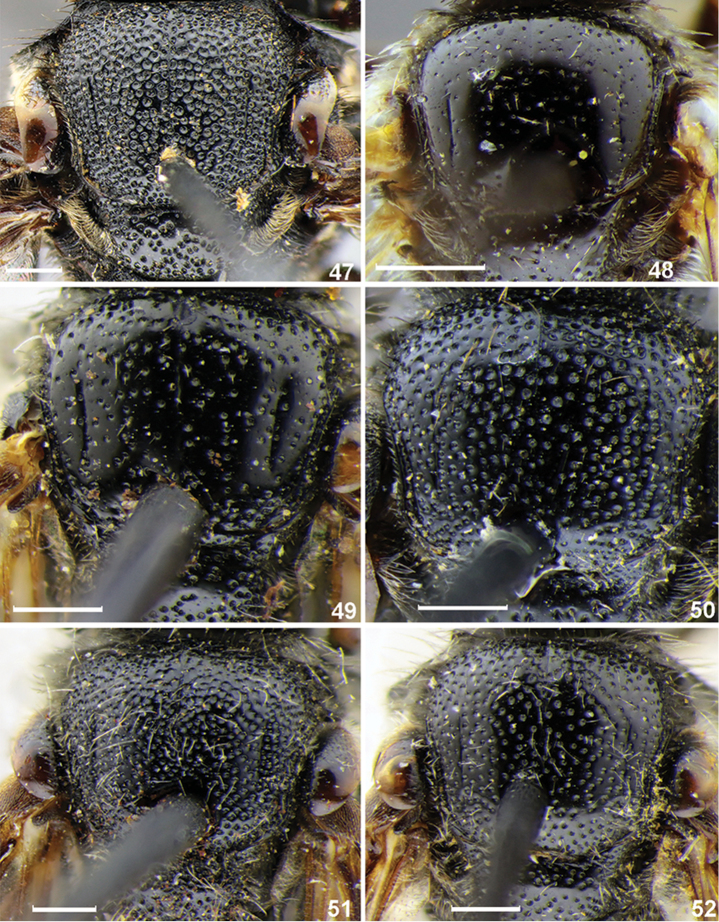
Scutum, females, dorsal view. **47***S.albilabris* (Fabricius) **48***S.pinguiculus* Pérez **49***S.intermedius* Blüthgen **50***S.nippon* Meyer **51***S.laticaudatus* Tsuneki **52***S.grahami* Cockerell. Scale bars: 0.5 mm.

**Figures 53–58.**
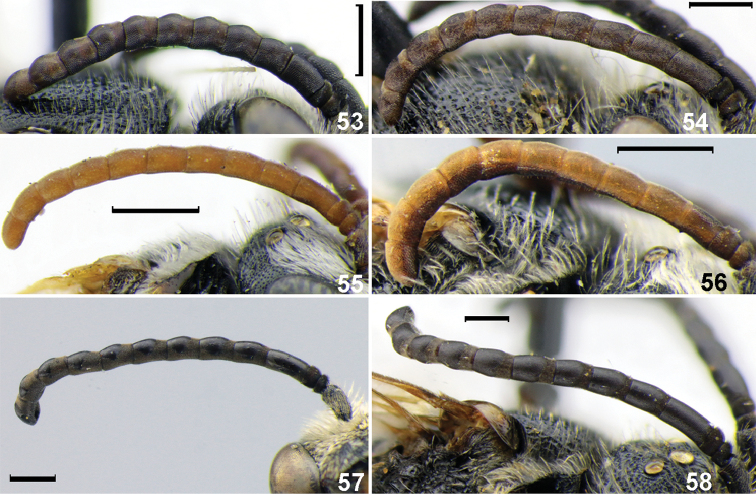
Antennae, males. **53***Sphecodespieli* Cockerell **54***S.kozlovi* Astafurova & Proshchalykin **55***S.pinguiculus* Pérez **56***S.intermedius* Blüthgen **57***S.gibbus* (Linnaeus) **58***S.nippon* Meyer. Scale bars: 0.5 mm.

## Discussion

In total, 33 species of *Sphecodes* are recorded from China (Table 1). Twenty-two of these species are Palaearctic and two species have a Palaearctic-Oriental range. For comparison, 37 species are known from Russia, 20 species of these from the Russian Far East (Astafurova and Proshchalykin 2014, 2017b) and 36 from Central Asia (Astafurova and Proshchalykin 2017a, Astafurova et al. 2018). In contrast, the Oriental fauna of the genus is poorly studied: only nine species are recorded from Oriental China, most of which are only known from type series, suggesting that further revision is necessary.

**Table 1.** Checklist of the *Sphecodes* species of China including distribution by provinces.

	Species	Province	Published data	Type of areal
1	*S.albilabris* (Fabricius, 1793)	Gansu, Liaoning, Inner Mongolia, Shanxi	first record	P
2	*S.alternatus* Smith, 1853	Xinjiang, Gansu	first record	P
3	*S.chinensis* Meyer, 1922	China (exactly locality is unknown)	Meyer 1922	?
4	*S.crassus* Thomson, 1870	Inner Mongolia, Shanxi	first record	P
5	*S.cristatus* Hagens, 1882	Xinjiang, Inner Mongolia, Ningxia, Liaoning, Hebei, Shandong, Shanxi, Shanxi, Heilongjiang, Jilin, Beijing, Tianjin	Meyer 1922, Blüthgen 1927, Ascher and Pickering 2018, current data	P
6	*S.ephippius* (Linné, 1767)	Xinjiang	first record	P
7	*S.ferruginatus* Hagens, 1882	Shanxi, Beijing	first record	P
8	*S.formosanus* Cockerell, 1911	Taiwan	Cockerell 1911	O
9	*S.galeritus* Meyer, 1927	Guandong	Meyer 1927	O
10	*S.gibbus* (Linnaeus, 1758)	Xinjiang	Meyer 1920, current data	P
11	*S.geoffrellus* (Kirby, 1802)	Shanxi, Inner Mongolia	first record	P
12	*S.grahami* Cockerell, 1922	Sichuan, Shanghai; Hebei, Shaanxi, Shanxi, Jilin, Jiangsu, Anhui, Zhejang, Xizang, Guandong, Yunnan	Cockerell 1922, 1931, Ascher and Pickering 2018, current data	PO
13	S. *howardi* Cockerell, 1922	Guandong	Cockerell 1922, Blüthgen 1924	O
14	*S.intermedius* Blüthgen, 1923	Gansu	first record	P
15	*S.kershawi* Perkins, 1921	Guandong	Meyer 1927	O
16	*S.kozlovi* Astafurova & Proshchalykin, 2015	Inner Mongolia, Ningxia, Shanxi	first record	P
17	*S.laticaudatus* Tsuneki, 1983	Hebei	first record	P
18	*S.laticeps* Meyer, 1920	Taiwan	Meyer 1920	O
19	*S.longulus* Hagens, 1882	Gansu, Shanxi, Hebei, Inner Mongolia	Blüthgen 1934, current data	P
20	*S.manchurianus* Strand & Yasumatsu, 1938	Liaoning	Strand and Yasumatsu 1938	P
21	*S.monilicornis* (Kirby, 1802)	Heilongjiang	first record	P
22	*S.nippon* Meyer, 1922	Gansu, Inner Mongolia, Shaanxi, Heilongjiang, Beijing, Gansu	Blüthgen 1934, current data	P
23	*S.nurekensis* Warncke, 1992	Xinjiang	first record	P
24	*S.olivieri* Lepeletier de Saint-Fargeau, 1825	Xinjiang, Gansu	first record	P
25	*S.pieli* Cockerell, 1931	Sichuan, Shanghai, Shanxi, Hebei, Beijing , Zhejiang, Jiangsu	Cockerell 1931, Ascher and Pickering 2018, current data	PO
26	*S.pinguiculus* Pérez, 1903	Gansu, Inner Mongolia	first record	P
27	*S.pectoralis* Morawitz, 1876	Xinjiang, Gansu	first record	P
28	*S.pellucidus* Smith, 1845	Xinjiang, Sichuan, Gansu	Blüthgen 1924, Meyer 1922, 1925, current data	P
29	*S.sauteri* Meyer, 1925	Taiwan	Meyer 1925	O
30	*S.scabricollis* Wesmael, 1835	Qinghai, Zhejiang, Shaanxi, Heilongjiang, Beijing	first record	P
31	*S.takaensis* Blüthgen, 1927	Taiwan	Blüthgen 1927	O
32	*S.tertius* Blüthgen, 1927	Guandong	Ascher and Pickering 2018	O
33	*S.turanicus* Astafurova & Proshchalykin, 2017	Gansu	first record	P

P – Palaearctic species; O – Oriental species; PO – Palaearctic and Oriental species

The majority of the Palaearctic Chinese *Sphecodes* is composed of 14 widespread Trans-Palaearctic or Euro-Asian species. Of them, eight species are distributed from Europe to the Russian Far East, Japan and the eastern provinces of China (*S.albilabris*, *S.crassus*, *S.cristatus*, *S.ferruginatus*, *S.geoffrellus*, *S.longulus*, *S.monilicornis*, and *S.scabricollis*). One species, *S.pellucidus*, occurs in the Russian Far East, but is rare in the East Palaearctic and has not yet been found in eastern China. Five species are distributed from Europe to Siberia and are, as expected, recorded in north-west China (*S.alternatus*, *S.gibbus*, *S.ephippius*, *S.pinguiculus*, *S.intermedius)*.

The other eight Palaearctic species have smaller distributional ranges. *Sphecodesolivieri* is found in semi-desert and desert habitats of the Western Palaearctic, including Xinjiang and Gansu within China. Three species, *S.nurekensis, S.pectoralis* and *S.turanicus*, are desert and steppe Irano-Turanian species distributed in Central Asia which also occur in Xinjiang and Gansu. Four species, *S.kozlovi*, *S.laticaudatus*, *S.manchurianus*, and *S.nippon* are East Palaearctic species, not found farther west than 90°E.

None of the above Palaearctic species are recorded below 30°N. However, two species, *S.grahami* and *S.pieli*, have an inter-realm range and are distributed in the East Palaearctic well as Oriental China. More study is necessary to revise the Oriental *Sphecodes*.

## Supplementary Material

XML Treatment for Sphecodes albilabris

XML Treatment for Sphecodes alternatus

XML Treatment for Sphecodes crassus

XML Treatment for Sphecodes cristatus

XML Treatment for Sphecodes ephippius

XML Treatment for Sphecodes ferruginatus

XML Treatment for Sphecodes geoffrellus

XML Treatment for Sphecodes gibbus

XML Treatment for Sphecodes grahami

XML Treatment for Sphecodes intermedius

XML Treatment for Sphecodes kozlovi

XML Treatment for Sphecodes laticaudatus

XML Treatment for Sphecodes longulus

XML Treatment for Sphecodes manchurianus

XML Treatment for Sphecodes monilicornis

XML Treatment for Sphecodes nippon

XML Treatment for Sphecodes nurekensis

XML Treatment for Sphecodes olivieri

XML Treatment for Sphecodes pectoralis

XML Treatment for Sphecodes pellucidus

XML Treatment for Sphecodes pieli

XML Treatment for Sphecodes pinguiculus

XML Treatment for Sphecodes scabricollis

XML Treatment for Sphecodes turanicus
